# Physiological Impacts of a Newly Discovered Trematode Parasite on Its Host, the Bay Scallop (*Argopecten irradians*)

**DOI:** 10.1002/ece3.73389

**Published:** 2026-04-07

**Authors:** Hailea F. H. Boggess, Connor R. Brainard, Brian Smith, Ami E. Wilbur, Julia C. Buck

**Affiliations:** ^1^ Department of Biology and Marine Biology University of North Carolina Wilmington Wilmington North Carolina USA; ^2^ Shellfish Research Hatchery, University of North Carolina Wilmington Wilmington North Carolina USA; ^3^ Center for Marine Science, University of North Carolina Wilmington Wilmington North Carolina USA

**Keywords:** bivalves, castration, clearance rate, condition index, trematodes

## Abstract

Parasites have the potential to impact aquaculture by degrading product quality, increasing mortality, and reducing fecundity. Previous research has identified a novel trematode parasite, *Saccularina* sp., infecting the gills of wild and cultured populations of bay scallops, *Argopecten irradians* , that inhabit the North Carolina coast and the Gulf Coast of Florida. However, the physiological impacts of *Saccularina* sp. on its scallop host are currently unknown. We quantified the effects of *Saccularina* sp. infection on clearance rate (an indicator of feeding potential), condition, and reproduction. Visually (to the naked eye) infected bay scallops exhibited lower clearance rates, had smaller muscle and gonad weights relative to shell size, and showed reduced fecundity compared to their visually uninfected counterparts. However, when scallops spawned successfully, there was no significant difference between visually infected and visually uninfected scallops in the proportion of embryos developing into D‐stage larvae. Overall, this study shows that *Saccularina* sp. reduces the metabolic energy available to *A. irradians* , resulting in decreased size, meat yield, and fecundity, with negative implications for aquaculture and presently‐depleted wild populations of bay scallops.

## Graphical abstract

A novel parasite, *Saccularina* sp., has been observed infecting the gills of bay scallops, *Argopecten irradians* , inhabiting the North Carolina coast and the Gulf Coast of Florida. Previous research has identified *Saccularina* sp. as a well‐established parasite in both wild and aquaculture populations of bay scallops throughout North Carolina, but the physiological impacts of *Saccularina* sp. on its bay scallop host is currently unknown. In this manuscript, we performed novel experiments to quantify the effects of *Saccularina* sp. infection through clearance rate trials, condition analyses, and spawning experiments.

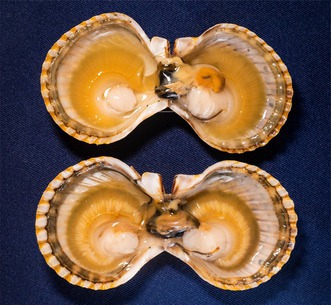

## 1 Introduction

*Saccularina* sp. is a newly discovered trematode in the family Didymozoidae that has been found infecting the gills of its first intermediate host, the bay scallop ( *Argopecten irradians* ; Lamarck, 1819), along the coast of North Carolina and the Gulf Coast of Florida (Boggess et al. 2024). Both cultured and wild bay scallops have been found to be infected with *Saccularina* sp., with peak prevalence ranging from 4.8%–22.4% (Boggess et al. 2024). Parasites harm their hosts through a range of pathological mechanisms, including trauma, nutrition robbing, toxin production, and interactions of the host immune/inflammatory responses, thereby impacting host growth, development, and fecundity (Roberts and Janovy 2000). However, little is known about the effects of *Saccularina* sp. (or indeed any didymozoid) on its first intermediate host's physiology.

Like other bivalves, bay scallops are suspension feeders. Water enters through their inhalant siphon and is filtered by the gills. Particulates caught in the gills are then transported to the labial palps where food is sorted and passed to the mouth. Researchers commonly use clearance rate, the rate by which water pumped by an animal is cleared of particulate matter via filtration, to quantify the effects of size, water temperature, and other factors on bivalve feeding behaviors (Bayne et al. 1993; Khosravi et al. 2023; Stier et al. 2015). Damage to or mechanical obstruction of gill feeding structures may lead to a decrease in host feeding potential. For example, infection of the blue mussel, *Mytilus edulis* (Linnaeus, 1758), by the trematode *Renicola roscovita* (Stunkard, 1932) reduced bivalve feeding efficiency, significantly reducing clearance rates (Khosravi et al. 2023; Stier et al. 2015). Since *R. roscovita* parasitizes the gills and labial palps of its host, researchers theorized that the observed clearance rate reduction may be due to localized host tissue damage and mechanical obstruction of flow. Since *Saccularina* sp. also infects host gill tissue, a similar effect may occur in infected *A. irradians* .

Measurements of body weight are commonly used to quantify effects of infection on host physiology (Paynter and Burreson 1991; Nagasawa and Nagata 1992). For bivalves, a frequent basis for such measurements is the dry weight of the animal tissue, which is determined by shucking, drying, and weighing the soft tissues contained in the shell. This can then be used to calculate other useful metrics such as condition index, a measure of the mass of an animal's dehydrated soft tissue relative to internal shell volume (Gabbott and Stephenson 1974; Lawrence and Scott 1982; Ranier and Mann 1992). An animal in optimal condition will have a high condition index, as a high proportion of tissue weight inside the shell is due to biomass rather than water. Since this measurement scales with overall animal size, it is useful in studying the effects of parasitism and other factors on the growth rates and health of bivalves. For example, common cockles, *Cerastoderma edule* (Linnaeus, 1758), infected by trematodes had lower condition indices than did uninfected cockles (de Montaudouin et al. 2012).

In addition to negatively impacting the overall health and growth of its host, parasites can have disproportionately negative effects on specific tissues, such as the adductor muscle in bivalves (Inglis et al. 2016). The size of a scallop's adductor muscle has a large impact on its marketability, as the muscle is the primary meat harvested in most parts of the world (Coleman 2021). The size of host gonads may also be of interest, as this is positively correlated with fecundity (Kasmini et al. 2019).

The time of year at which a bay scallop population spawns varies depending on latitude because temperature, salinity levels, and timing of phytoplankton blooms impact the timing of gametogenesis and spawning (Mackenzie 2008; Taylor and Capuzzo 1983). Bay scallops in Massachusetts spawn first in June and July, while Florida populations tend to spawn in October (Barber and Blake 1983). In North Carolina, gonadal growth peaks in August and September with subsequent spawning through November (Barber and Blake 1983; Sastry 1970).

Soon after fertilization (40–50 min), the embryo begins cleavage until a ciliated blastula forms about 5 h post‐fertilization (Leavitt et al. 2010). Approximately 17–48 h post‐fertilization, larvae develop into straight‐hinge veliger, or D‐stage (Leavitt et al. 2010). At this stage, the larva has developed its velum, the swimming and feeding organ. About 14 days post‐fertilization, the larvae settle and attach to substrate and develop into juvenile scallops approximately 60–120 days post fertilization (Mackenzie 2008).

Castration, a parasite strategy in which host fecundity is reduced or terminated entirely, is commonly employed by trematodes in their first intermediate hosts (Baudoin 1975; Hurd 2001). This strategy allows the parasite to access and consume host energy while maintaining or even increasing host longevity, thereby maximizing parasite fecundity (Lafferty and Kuris 2009). Oftentimes, trematodes inhabit the gonads of first intermediate hosts, acquiring energy by targeting reproductive energy or through direct consumption of gonadal tissue (Baudoin 1975; Faro et al. 2013; Hechinger et al. 2008; Valderrama et al. 2004). However, *Saccularina* sp. inhabits the afferent vessels of scallop gill tissue (Boggess et al. 2024) rather than the gonad, so if castration occurs, it would likely be attributable to indirect energy drain.

Here we test the effect of *Saccularina* sp. infection on bay scallop clearance rates, condition, and fecundity. We predict that *Saccularina* sp. infection will negatively impact the clearance rate, condition, and fecundity of bay scallops. This work will provide insights into the impacts of this novel parasite on bay scallop aquaculture and already‐dwindling wild populations.

## 2 Methods

### 2.1 Confirmation of Infection Status

**FIGURE 1:**
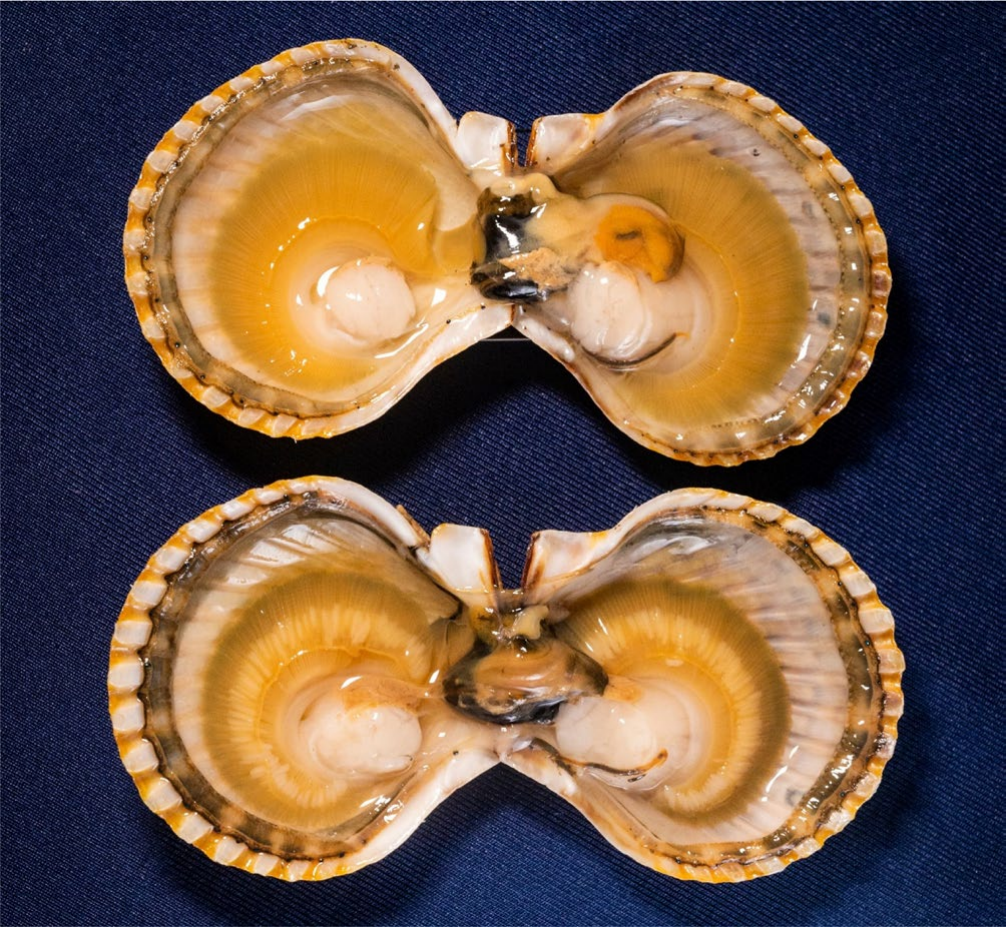
Uninfected (top) and *Saccularina* sp. infected (bottom) bay scallop ( *Argopecten irradians* ) showing expanded afferent vessels visible to the naked eye. Photo credit: Jeff Janowski.

Our investigations of clearance rates, condition, and fecundity rely on visual identification of infection. Once a *Saccularina* sp. infection has progressed beyond the trematode's pre‐patent period of 46 days, greatly expanded afferent vessels (which contain sporocysts) are readily visible to the naked eye (Figure 1) (Boggess et al. 2024). Thus, it is simple to determine a scallop's infection status non‐destructively by gently prying open the scallop (< 1 cm) and visually inspecting the gill tissue using a headlamp. Nevertheless, to confirm that infection status can be reliably determined visually, we conducted PCR analysis using didymozoid‐specific primers on gill samples dissected from 16 visually infected and 16 visually uninfected scallops raised on the UNCW Aquaculture Demonstration Lease in the Intracoastal Waterway behind Masonboro Island and sacrificed in December 2024 (Brainard 2024; Appendix 1).

### 2.2 Clearance Rates

Scallops used in clearance rate experiments were obtained from the same generational cohort spawned at the UNCW Shellfish Research Hatchery (SRH), grown on UNCW's Aquaculture Demonstration Lease, and collected in fall 2022 (29 visually infected and 29 visually uninfected). After being collected and all shell biofouling gently removed, study animals were kept in a holding tank within the SRH and were allowed to acclimate for at least 3 days. Water conditions were kept between 15°C and 18°C and at a salinity of ~30 ppt. Scallops were fed an algae mixture of *Tisochrysis lutea, Pavlova* sp., *Chaetoceros* sp., and *Thalassiosira* sp. and were regularly inspected for biofouling and mortalities.

Clearance rate trials were conducted using a static system. To encourage feeding and minimize the presence of feces, study animals were gently scrubbed immediately before trials to remove any excrement or biofouling on the exterior of their shells, and subsequently starved for 24 h. During this period, scallops were housed directly underneath the tank inflow for acclimation to moderate water agitation, which occurred during trials as a result of aeration.

While airflow was essential to mix algae during trials, high flow rates were found to stress the animals, causing them to close their shells and halt feeding. Preliminary trials were performed to determine the optimal airflow into the containers to allow for sufficient mixing of algae, while not causing any detectable alteration in scallop behavior. Airstones were placed on the far side of the container from the scallop to minimize disturbance, and human observation and handling during trials were minimized and done at a distance, when feasible.

**FIGURE 2:**
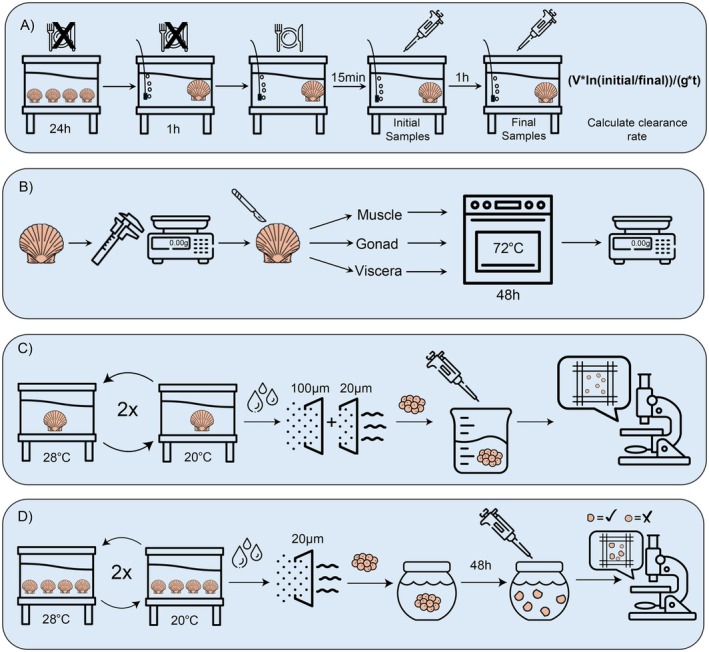
Schematic of experimental workflows for (A) Clearance rate trials (B) Condition analysis (C) Individual spawning trials and (D) Group spawning trials.

The first five trials included five visually infected and five visually uninfected *A. irradians* , and the last trial consisted of four of each group (Figure 2a). At the start of each trial, filtered seawater was used to rinse and then fill ten 5 L tanks up to 4 L; airflow to each tank was set to the previously‐described level, then a single scallop was added to each tank and allowed to acclimate for 1 h. After this acclimation period, an algal mixture at a concentration of 300,000 cells/mL was added, bringing total tank volume to 4.3 L. Source algae concentration was confirmed before each trial via Coulter Counter measurement.

Animals were allowed 15 min to acclimate to this disturbance (addition of algal mixture), then three water samples of roughly 30 mL each were taken from each tank for initial algal concentration counts. Animals were allowed to feed for 1 h, then three final water samples of the same volume were taken from each tank. Animals were then placed into individually labeled bags and frozen for condition analysis. Initial and final algal concentrations were calculated and then averaged for each animal. One visually infected specimen stayed closed during its trial and algae measurements show that this animal did not eat, so it was excluded from subsequent analyses.

### 2.3 Condition Analysis

Eighty‐eight visually infected and 70 visually uninfected scallops were collected in three separate sampling windows: 2019 (24 visually infected, 19 visually uninfected), 2022 (29 visually infected, 29 visually uninfected), and 2023 (35 visually infected, 22 visually uninfected) and were processed for condition analysis. With the exception of 14 visually infected and 14 visually uninfected scallops collected from deployments in Topsail Sound in 2023, all scallops were collected from deployments on the UNCW Aquaculture Demonstration Lease. All scallops were collected in the fall at 12–16 months of age.

Scallops were measured for shell dimensions, weighed whole, and tissues were dissected and separated into muscle, gonadal, and visceral (everything else) components (Figure 2b). Tissues were then dried at 72°C for 48 h and weighed. Total dry weights, condition index, and the proportion of dry weight that was made up of each of the tissue types were calculated (Gabbott and Stephenson 1974; Lawrence and Scott 1982; Pichaud et al. 2009).

### 2.4 Fecundity

All spawning experiments were performed at the UNCW SRH. Single spawn experiments were performed in the fall of 2022 and group spawn experiments were performed on two separate occasions: fall 2022 and fall 2023. Due to variations in group spawn methods, each year will be discussed as a distinct experiment. Scallops used in all spawning experiments were conditioned in the SRH in 400 L flow through tanks filled with filtered seawater, and fed a mixture of *Tisochrysis lutea*, *Pavlova* sp., *Chaetoceros* sp., and *Thalassiosira* sp. ad libitum to optimize gonadal development prior to spawning.

All scallops utilized in 2022 spawning experiments were sourced from different spawn groups from the previous year at the UNCW SRH. In 2022, single and group spawning experiments were completed over the course of 53 days using a randomized block design. To assess the effect of infection on egg quantity, individual spawn events were run on a total of 27 visually uninfected and 29 visually infected scallops (Figure 2c). Individuals were opportunistically collected from floating cages at the UNCW Aquaculture Demonstration Lease and brought into the SRH to adjust to hatchery conditions prior to spawning experiments. Depending on which block each scallop was spawned in, they experienced a conditioning period of 11–25 days leading up to the experiment. Each scallop was individually housed in a 6.31 L tank filled with 4 L filtered seawater and all tanks were partially submerged in a flow‐through table to help regulate water temperature. Spawning was triggered using thermal stimulation (Castagna 1975; Castagna and Duggan 1971; Li et al. 2011; Sastry 1963). Once visible spawning began, any subsequent water change for individual tanks was strained through 100‐μm and 20‐μm sieves to collect any eggs that had been released. Spawn time was measured from when the first individual showed signs of spawning (release of eggs or sperm) until the experiment was terminated and ranged from 284–310 (mean = 298.6) minutes depending on spawn block. Eggs produced from each individual were suspended in 4 L of filtered seawater and three 250 μL subsamples were collected and preserved in 750 μL 95% ethanol solution. The entire 1 mL egg suspension was transferred to a Sedgewick Rafter Counting Cell and eggs were counted under a compound microscope. The egg counts from the three subsamples were then averaged and the averages used to estimate the total number of eggs in the 4 L suspension.

To assess the effect of infection on larval development, scallops were spawned in groups of 4 (Figure 2d). All 2022 group spawns were performed using the same scallops that spawned individually in the experiment described above. Because of this, the bay scallops required a period of reconditioning (which ranged from 25–48 days). Bay scallops were gently pried open (< 1 cm) and their gonads visually inspected after reconditioning to ensure an available store of gametes for subsequent group spawning. Each spawn group was housed in a 6.31 L tank filled with 4 L of filtered seawater with another tank of the same size left empty for necessary temperature switches, and all tanks were partially submerged in a flow through seawater table. Similarly to the individual spawning procedure, thermal stimulation was used to provoke spawning and spawn time was measured from when the first spawn group showed signs of spawn behavior until the end of the spawn block. Group spawn time for 2022 and 2023 experiments ranged from 251–312 (mean = 281.5) minutes and 230–260 (mean = 240.8) minutes. Embryos were collected on a 20‐μm sieve, rinsed, and suspended in 4 L of filtered sea water to estimate embryo number using the same methods described above. Embryos were stocked at 20/mL in 15 L buckets, which is consistent with stocking densities used in shellfish hatcheries. Bucket total volume varied between experimental blocks to ensure consistent larval density. Once tanks were set up under gentle aeration and partially submerged in a water bath to maintain a constant temperature (26°C), three 250 μL subsamples were collected and preserved in 750 μL 95% ethanol solution. Feeding was initiated at 24‐h post‐fertilization and development proceeded until approximately 48 h post‐fertilization, when bay scallop larvae are expected to reach D‐stage (Li et al. 2011). Larvae were then collected by filtering the contents of each tank through a 40‐μm mesh filter, rinsed, and suspended in a total volume of 4 L. Three 250 μL subsamples were collected and preserved in 750 μL 95% ethanol solution. Samples collected at the beginning and end of the experiment were counted and averaged using the same method described for single spawning experiments. Any larvae that did not reach D‐stage by the 48 h sample collection were deemed dead or unviable and were not included in the estimate.

Bay scallops used in 2023 group spawn experiments were a mixture of wild and hatchery sourced individuals and were housed under hatchery conditions 0–29 days prior to spawning. Spawning was again achieved using thermal stimulation, and the process of water changes and filtration was the same as those in the 2022 group spawn experiment. Once spawning was complete, the embryos were sieved, rinsed, and suspended in 2–4 L of filtered sea water to estimate larval production using the same methods described above. Embryos were stocked in 15 L buckets at densities ranging from 0.2–8/mL. Tanks were held in a water bath to maintain optimal temperature (26°C) under constant aeration, fed after 24 h, and left to develop until 48 h post‐fertilization. Larvae were then collected by filtering the contents of each tank through a 20‐μm mesh filter, rinsed, and suspended in a total volume of 50 mL. Three 250 μL subsamples were collected and preserved in 750 μL 95% ethanol solution. Larval samples were counted and estimated using the same method described for single spawning experiments.

### 2.5 Data Analysis

Clearance rates were calculated using the following equation: $\left(V*\ln\left(\text{initial}/\text{final}\right)\right)/\left(g*t\right)$, where *V* is water volume, *g* is dry tissue weight, *t* is time (1 h) and initial and final are algal concentrations at the beginning and end of trials. A two‐way ANOVA with replication was run in R version 4.3.1 to determine if infection status and trial number had a significant effect on clearance rates.

Condition indices were calculated as follows: $\begin{array}{c} ((\mathrm{Dry}\;\text{tissue weight})*100)/(\text{Total scallop}\;\mathrm{wet}\;\text{weight}-\mathrm{Dry}\;\text{shell}\; \end{array}$ $\begin{array}{c} \text{weight}) \end{array}$. Unpaired *t*‐tests were performed to determine whether infection status affected condition index, overall dry tissue weight, and shell height. To isolate the effect of infection status on weights of different tissues, ANCOVAs were performed using the dry weight of the tissue in question as the treatment and the shell height of the animal as the covariate. Individual tests were performed for muscle, gonad, and visceral tissue.

To analyze single spawn experiments, we used the package lme4 to perform a GLMM with a negative binomial distribution using egg quantity as the dependent variable, infection status as a fixed independent variable, and duration spawned, time conditioned, and scallop height as random independent variables. Due to the use of multiple variables in this model, we utilized a model selection approach to test all biologically relevant variable combinations and ranked models based on their Akaike Information Criterion (AIC) (Akaike 1974). To analyze both group spawn experiments, conversion rate to D‐stage larvae was calculated using initial and final larvae quantity to assess percent survival. We then used the mean conversion rate of infected and uninfected groups to perform a one‐tailed, unpaired *t*‐test.

## 3 Results

### 3.1 Confirmation of Infection Status

The infection status of all 32 samples was correctly identified using PCR. Visually infected scallops amplified as expected, whereas all visually uninfected scallops failed to amplify. Therefore, we maintain that infection status can be reliably determined visually. However, we acknowledge the possibility that one or more scallops used in our investigations of clearance rates, condition, or fecundity might have been harboring a pre‐patent infection. We therefore refer to scallops as “visually infected” or “visually uninfected” and note that physiological impacts of pre‐patent infections are likely to be minimal.

### 3.2 Clearance Rates

**FIGURE 3:**
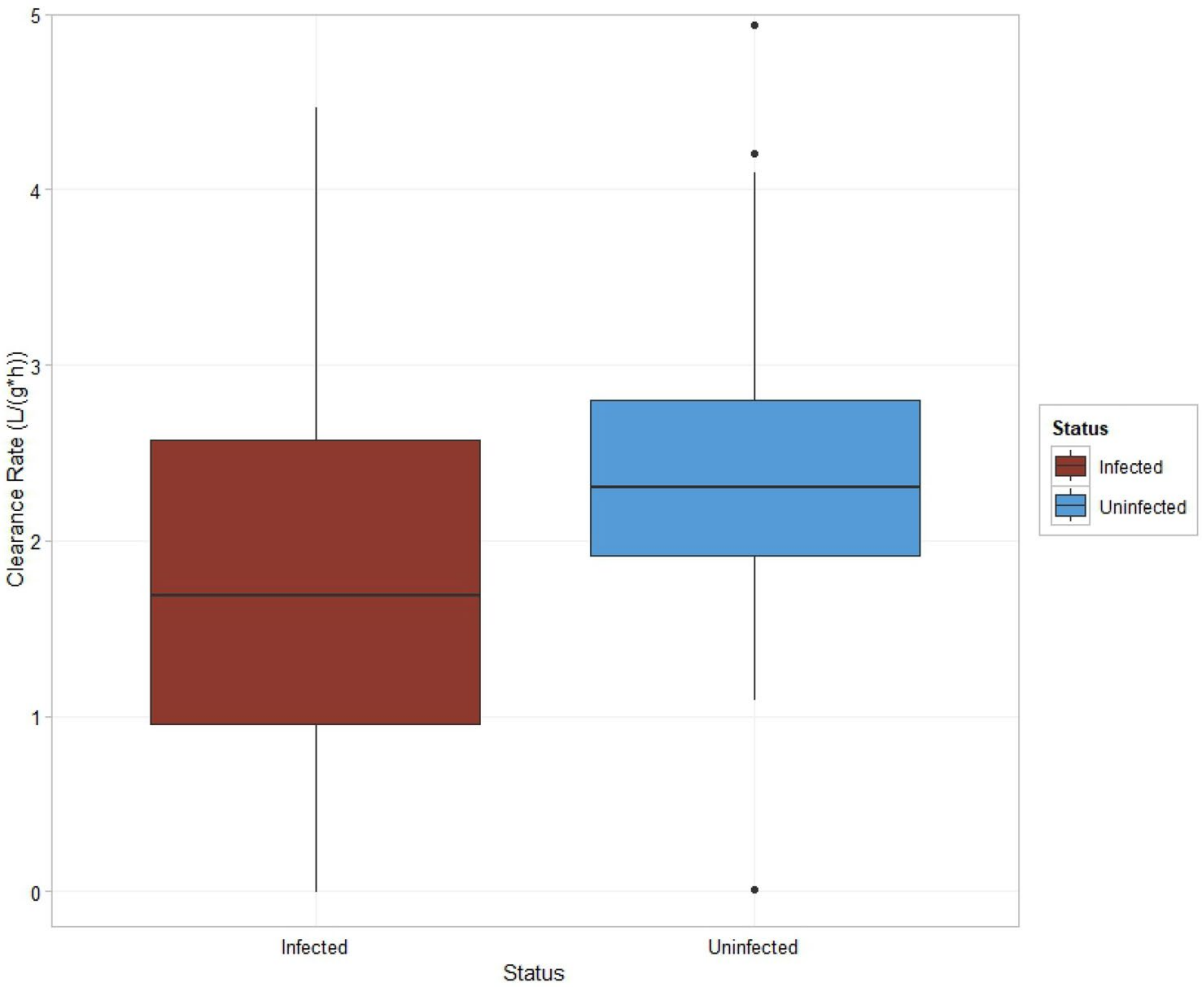
Boxplot of clearance rates of bay scallops ( *Argopecten irradians* ) as a function of *Saccularina* sp. infection status: Infected (left) and uninfected (right) (*p* = 0.037).

Of the 29 visually infected and 29 visually uninfected scallops used in clearance rate trials, one visually infected scallop remained closed and did not show signs of feeding, so it was not included in statistical analyses. The average clearance rate and standard error was 2.44 ± 0.19 L/(g*h) for the uninfected treatment, and 1.82 ± 0.25 L/(g*h) for the infected treatment (Figure 3). A two‐way ANOVA determined that both infection status (*p* = 0.037) and trial number (*p* = 0.025) had a significant effect on clearance rate, and that there was no interaction effect between the two variables (*p* = 0.60).

### 3.3 Condition Analysis

**FIGURE 4:**
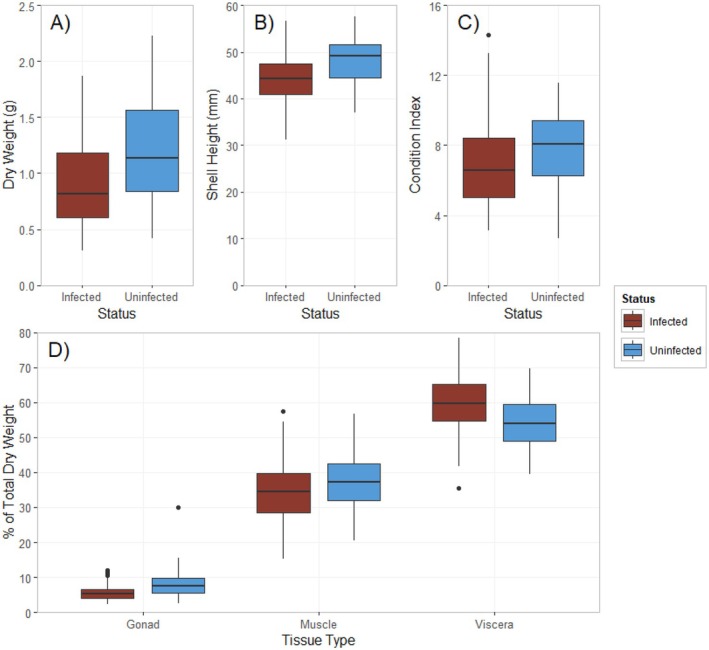
(A) Boxplot of *Argopecten irradians* dry tissue weights by *Saccularina* sp. infection status: Infected (left) and uninfected (right) (*p* = 9.9e‐7). (B) Boxplot of *Argopecten irradians* heights by infection status (*p* = 4.7e‐6). (C) Boxplot of condition indices of *Argopecten irradians* by infection status (*p* = 0.012). (D) Box plot showing a tissue‐specific comparison of bay scallops ( *Argopecten irradians* ) by infection status (gonad *p* = 0.00021, muscle *p* = 0.00305, viscera *p* = 0.32).

Visually infected scallops had an average total dry tissue weight of 0.90 ± 0.04 g, while the average for visually uninfected scallops was 1.22 ± 0.05 g (Figure 4a). This difference was confirmed to be significant via an unpaired *t*‐test (*p* = 9.9e‐7). A similar pattern was observable through shell heights, with an average height of 44.11 ± 0.55 mm for visually infected scallops, and 48.00 ± 0.60 mm for visually uninfected scallops (Figure 4b). This difference was also confirmed to be significant via an unpaired *t*‐test (*p* = 4.7e‐6). The average condition index of visually infected scallops was 6.91 ± 0.26 compared to 7.87 ± 0.26 for the visually uninfected scallops (Figure 4c). This difference was also significant (*p* = 0.012).

The average proportion of body mass made up of viscera was 59.79% ± 0.89% for visually infected specimens and 54.10% ± 0.83% for the visually uninfected specimens (Figure 4d). Gonadal tissue comprised 5.56% ± 0.23% of visually infected specimen weight and 8.26% ± 0.48% for visually uninfected specimens (Figure 4d). Adductor muscle tissue proportions made up 34.66% ± 0.91% of visually infected and 37.64% ± 0.91% of visually uninfected tissue weights (Figure 4d). An ANCOVA on tissue weights using scallop height as the covariate to isolate the effect of visual infection status on weights of dry tissue types from the effect of overall scallop size found that scallop height had a significant effect on the weights of gonad, muscle, and visceral tissues (*p* < 1.0e‐8 for all). Visual infection status had a significant effect on gonad and muscle tissue weights (*p* = 2.1e‐4 and *p* = 0.00305, respectively), but not on visceral tissue weights (*p* = 0.32).

### 3.4 Individual Spawning

**FIGURE 5:**
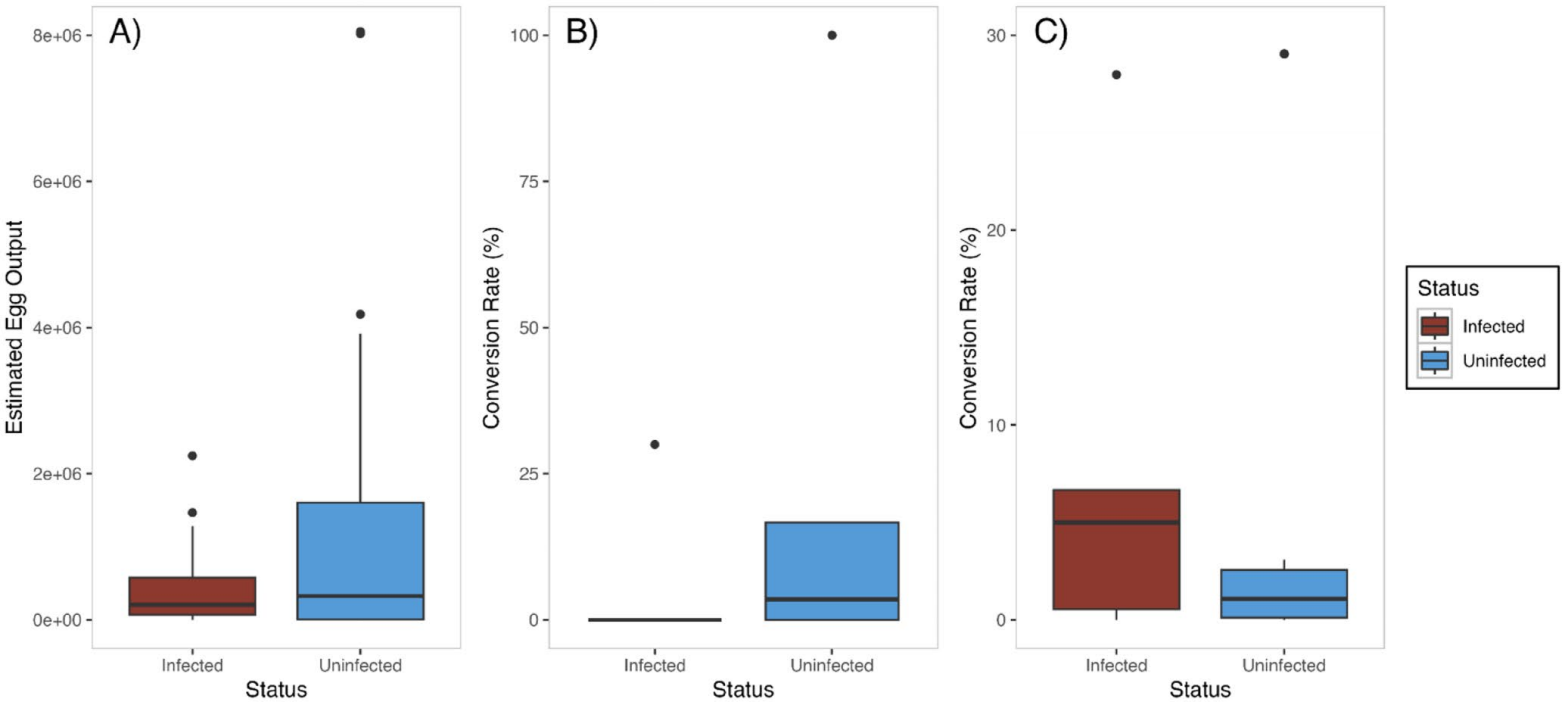
(A) Boxplot of estimated egg output for individually spawned bay scallops ( *Argopecten irradians* ) as a function of *Saccularina* sp. infection status: Infected (left) and uninfected (right) (*p* = 0.035). (B) Boxplot of estimated conversion rate (%) of bay scallop ( *Argopecten irradians* ) larvae for 2022 group spawning experiments as a function of infection status (*p* = 0.16). (C) Boxplot of estimated conversion rate (%) of bay scallop ( *Argopecten irradians* ) larvae for 2023 group spawning experiments as a function of infection status (*p* = 0.29).

The height of bay scallops at the time of spawning ranged between 38.9–60.7 mm with a mean of 47.8 ± 0.55 mm. Spawn time ranged from 284–310 min with a mean of 298.6 min. AIC scores for all models were very similar, but the model of best fit included only visual infection status, suggesting that time conditioned, time spawned, and spawning block had no significant impact on egg production. GLM results indicate that visual infection status has a significant impact on egg output, with visually infected bay scallops producing fewer eggs than visually uninfected individuals (*p* = 0.035, Figure 5a).

### 3.5 Group Spawning

Shell height of bay scallops at the time of spawning ranged between 40.0–63.2 mm with a mean of 51.4 ± 0.66 mm in 2022 and 37.9–56.4 mm with a mean of 48.3 ± 0.49 mm in 2023. The 2022 group spawn experiment had a total of 12 spawning groups, but only 11 groups successfully spawned: 6 visually infected and 5 visually uninfected. The 2023 group spawn experiment had a total of 18 spawning groups, but only 13 groups successfully spawned: 5 visually infected and 8 visually uninfected. In 2022, mean conversion rate was observationally higher for visually uninfected groups (24.03% ± 17.56%) compared to visually infected groups (5% ± 5%). However, *t*‐test results showed that there was no clear difference in conversion rate to D‐stage larvae in visually infected versus visually uninfected groups (*p* = 0.16, Figure 5b). In 2023, the inverse relationship was found. Mean conversion rate was observationally lower in visually uninfected groups (4.6% ± 3.51%) compared to visually infected groups (8.04% ± 5.14%), but again this difference was not found to be statistically significant (*p* = 0.29, Figure 5c).

## 4 Discussion

Overall, our results demonstrate significant negative impacts of *Saccularina* sp. infection on *Argopecten irradians* feeding, condition, and reproduction. Previous research has found that, after controlling for size, smaller bivalves typically exhibit higher clearance rates than larger conspecifics (Jacobs et al. 2015). Given that infected bay scallops had lower average dry weights and shell sizes than uninfected scallops, one might expect infected individuals to exhibit higher clearance rates, all else being equal. Instead, we found that infected scallops had lower clearance rates than uninfected scallops, demonstrating that infection reduces filter feeding. At least in part, this is likely due to mechanical disruption by the parasite‐induced expansion of the host's afferent vessels within the gill (Boggess et al. 2024).

Even though scallops were starved in preparation for clearance rate trials, some individuals still produced feces and pseudofeces. This has the potential to lower clearance rate measurements by increasing the overall amount of suspended particulates and can even result in negative clearance rates if individuals don't feed within the trial. While the only negative clearance rate in this trial was deemed an “inactive” individual and excluded from analyses, production of feces and pseudofeces may have lowered the calculated clearance rates of other individuals. However, this phenomenon would be evenly distributed across infected and uninfected individuals, so it would be unlikely to influence results. In order to prevent such a dampening effect in future studies, we recommend a longer fasting period or the use of a continuous flow experiment with a built‐in filter for feces and pseudofeces (Leverone et al. 2007).

Condition analyses show that *Saccularina* sp. infection negatively impacts scallop tissue weight, height, condition index, and development of muscle and gonad tissue. This result may be partially explained by reduced feeding efficiency of infected scallops, as seen in our clearance rate experiment, although energy drain by *Saccularina* sp. could also contribute. Given that we were unable to separate parasite tissue from visceral tissue, we were unable to draw conclusions about the parasite's effect on this tissue type. Additionally, because we quantified infection status rather than intensity, which was beyond the scope of our study, individuals may differ in the degree of infection progression, and therefore could differ in the degree of energy drain from the parasite.

Significantly lower gonad weight in infected scallops supports the results of our individual spawning experiment showing that *Saccularina* sp. infection negatively impacts, but does not completely eliminate, egg production. Previous research illustrates similar castrating effects of trematodes in first intermediate hosts (Lafferty 1993; Sorensen and Minchella 2001), but the vast majority of research examines trematodes that infect the gonadal tissue of their first intermediate hosts (Faro et al. 2013; Valderrama et al. 2004). In contrast, *Saccularina* sp. infects the afferent vessels of its first intermediate host (Boggess et al. 2024), so impacts on fecundity seem more likely to be caused by overall energy drain, similar to some other castrating parasites (Calado et al. 2008; Sherman and Curran 2015). Once established, trematode infections in first intermediate hosts are generally not able to be cleared (Erasmus 1972). We found that all visually infected bay scallops remained infected with *Saccularina* sp., suggesting no successful immune response to infection. A longer duration of infection could have more severe consequences on reproduction and body condition. Examining how the timing of infection impacts scallop reproduction was beyond the scope of this study, but would be worth examining in the future. In some cases, a trematode's first intermediate host will engage in a reproductive burst, often referred to as fecundity compensation, soon after infection (Hurd 2001; Lafferty and Kuris 2009). However, many trematodes, including those closely related to *Saccularina* sp., are capable of evading detection, and therefore elimination, by the host's immune system (Dang et al. 2013; Kawasaki et al. 2013; Mastore and Brivio 2008; Schmid‐Hempel 2008). *Saccularina magnacetabula* was found to evade the host immune system (Dang et al. 2013) by using highly host‐specific surface carbohydrates to avoid being recognized and encapsulated by host hemocytes (Kawasaki et al. 2013). Based on these results, it seems unlikely that bay scallops would exhibit fecundity compensation when infected with *Saccularina* sp., but further investigation into this mechanism is warranted.

Both group spawn experiments demonstrated that parental *Saccularina* sp. infection has no clear impact on early larval development of offspring. During conditioning in the hatchery, scallops were fed ad libitum. Therefore, it is possible that visually infected scallops still had enough energy to provision their gametes, despite energy drain due to infection with *Saccularina* sp. (Hall et al. 2007; Hurd 2001). In the 2022 group spawn experiment, there was an observationally higher conversion rate in visually uninfected groups, but this is likely due to one spawn group that had a 100% conversion rate after 48 h. In the 2023 group spawn experiment, the opposite trend was observed, though the difference between visually infected and visually uninfected individuals was statistically insignificant in both experiments. Given that we used infection status rather than intensity, visually infected individuals used in the 2023 group spawn experiment may have differed from the 2022 groups in degree of infection, which could contribute to differences in reproductive output. Tracking any progression of infection was outside the scope of our work, but studying the rate of parasite growth within the afferent vessel would be worth investigating. Differences between the 2022 and 2023 experiments might also be attributable to sample size and multiple unsuccessful spawning groups, particularly in the visually infected treatment. Any spawning groups that failed to release eggs and sperm during the spawning window were excluded from analyses, which resulted in 6 visually infected and 5 visually uninfected groups in 2022 and 5 visually infected and 8 visually uninfected groups in 2023. Because there were fewer visually infected groups than visually uninfected groups in 2023, any successful gamete production in the visually infected treatment would have a greater impact on the results. Furthermore, in both spawning years, many spawn groups in both visually infected and visually uninfected treatments had a 0% conversion rate. Our sample sizes were limited by the availability of infected individuals. Single and group spawn experiments should be replicated with larger sample sizes, using different genetic lines of bay scallops, and under different conditions to examine the impacts of hatchery conditions on reproductive success.

## 5 Conclusion

Our results indicate that *Saccularina* sp. infection negatively impacts clearance rates, overall scallop size, muscle and gonad weight, and egg production. However, when visually infected scallops spawned successfully, there was no significant difference in larval development when compared to visually uninfected groups. The detrimental effects of *Saccularina* sp. on bay scallops present challenges for the development of the bay scallop aquaculture industry and may also impact the recovery of wild populations. Expanding on this research is essential for further elucidating the impacts of this novel parasite on bay scallops and the implications for economic and human dimensions of the bay scallop industry.

## Author Contributions

**Hailea F. H. Boggess:** conceptualization (equal), formal analysis (equal), investigation (equal), methodology (equal), writing – original draft (equal), writing – review and editing (equal). **Connor R. Brainard:** conceptualization (equal), formal analysis (equal), investigation (equal), methodology (equal), writing – original draft (equal), writing – review and editing (equal). **Brian Smith:** investigation (supporting), methodology (supporting). **Ami E. Wilbur:** conceptualization (equal), funding acquisition (supporting), methodology (supporting), resources (lead), supervision (equal), writing – review and editing (supporting). **Julia C. Buck:** conceptualization (equal), funding acquisition (lead), methodology (supporting), resources (supporting), supervision (equal), writing – original draft (supporting), writing – review and editing (equal).

## Funding

This work was supported by North Carolina Sea Grant, North Carolina State University, NA22OAR4170109.

## Conflicts of Interest

The authors declare no conflicts of interest.

## Supporting information


**Appendix S1:** Supporting Information.

## Data Availability

The data that support the findings are available on Dryad at https://doi.org/10.5061/dryad.q573n5txt.
