# Supplementary material for: Physiological Impacts of a Newly Discovered Trematode Parasite on Its Host, the Bay Scallop (Argopecten irradians)
Source: Ecol Evol. 2026 Apr 7;16(4):e73389. doi: 10.1002/ece3.73389 (PMC13057426; doi:10.1002/ece3.73389)
Supplement: Supplementary file 1 — Appendix S1: Supporting Information. [file ECE3-16-e73389-s001.docx]

**Appendix 1**

To confirm that infection status can be reliably determined visually, we conducted PCR on the *cox1* gene locus. Gill samples were dissected from sixteen visually infected and sixteen visually uninfected scallops. These samples were kept at -20℃ until DNA extraction and genetic analysis. Total genomic DNA extraction was performed on gill tissue using the PureGene DNA extraction protocol (Qiagen, Inc) with reagent volumes proportionally reduced for use with small tissue samples. Once extracted, DNA pellets were resuspended with 35µl of deionized water. DNA concentrations were then determined via a NanoDrop One microvolume spectrophotometer and standardized to 100ng/µl with the addition of deionized water to 5µl of concentrate. DNA vials were stored at -20℃ until polymerase chain reaction (PCR) was performed.

Amplification of a 913-base pair section of the cox1 mitochondrial gene locus was performed using Didymozoid-specific forward primer 5’ TTTTTTGGGCATCCTGAGGTTTAT 3’ and reverse primer 5’ CAACAAATCATGATGCAAAAGG 3’ (Mladineo et al., 2010). Each 25µl reaction contained 1µl of diluted DNA template, 15.4µl sterile deionized water, 5µl 5x PCR buffer, 0.5µl 10mM dNTPs, 0.5µl cox1 forward primer, 0.5µl cox1 reverse primer, and 5U/µl GoTaq Flexi DNA polymerase (Promega). Each DNA sample underwent PCR alongside positive and negative controls. For the positive control, a DNA sample that was previously confirmed for parasite presence was used. The negative sample consisted of the PCR mixture but without any DNA template.

Parameters for PCR cycling in an Eppendorf Mastercycler Nexus Gradient Thermal Cycler consisted of the following steps: an initial 30s denaturation period at 94℃ followed by 35 cycles of 94℃ for 30s, 56℃ for 90s, 72℃ for 90s, and a single extension of 72℃ for 10 min at the end (Mladineo et al., 2010). Presence/absence of Cox1 amplification was confirmed via gel electrophoresis on a 1% agarose gel stained with ethidium bromide. UV radiation was used via a 15 FisherBiotech FBTIV-88 Transilluminator to show fluorescent DNA bands for each sample, and to make sure that the negative control did not appear.
